# A novel isoform of Homeodomain-interacting protein kinase-2 promotes YAP/TEAD transcriptional activity in NSCLC cells

**DOI:** 10.18632/oncotarget.27871

**Published:** 2021-02-02

**Authors:** Yuyuan Dai, Hiroyuki Kyoyama, Yi-Lin Yang, Yucheng Wang, Shu Liu, Yinghao Wang, Jian-Hua Mao, Zhidong Xu, Kazutsugu Uematsu, David M. Jablons, Liang You

**Affiliations:** ^1^Thoracic Oncology Laboratory, Department of Surgery, Comprehensive Cancer Center, University of California, San Francisco, CA, USA; ^2^Model Animal Research Center of Nanjing University, Nanjing, Jiangsu, China; ^3^Department of Pulmonary Medicine, Saitama Medical Center, Saitama Medical University, Kawagoe, Saitama, Japan; ^4^Biological Systems and Engineering Division, Lawrence Berkeley National Laboratory, Berkeley, CA, USA; ^*^These authors contributed equally to this work

**Keywords:** Homeodomain Interacting Protein Kinase 2 (HIPK2), non-small cell lung cancer (NSCLC), yes-associated protein (YAP), HIPK2 isoform, 4,5,6,7-tetrabromo-2-(1H-imidazol-2-yl)isoindoline-1,3-dione (TBID)

## Abstract

Homeodomain-interacting protein kinase-2 (HIPK2) can either promote or inhibit transcription depending on cellular context. In this study, we show that a new HIPK2 isoform increases TEAD reporter activity in NSCLC cells. We detected HIPK2 copy number gain in 5/6 (83.3%) NSCLC cell lines. In NSCLC patients with high HIPK2 mRNA expression in the Human Protein Atlas, the five-year survival rate is significantly lower than in patients with low expression (38% vs 47%; *p* = 0.047). We also found that 70/78 (89.7%) of NSCLC tissues have moderate to strong expression of the N-terminal HIPK2 protein. We detected and cloned a novel HIPK2 isoform 3 and found that its forced overexpression promotes TEAD reporter activity in NSCLC cells. Expressing HIPK2 isoform 3_K228A kinase-dead plasmid failed to increase TEAD reporter activity in NSCLC cells. Next, we showed that two siRNAs targeting HIPK2 decreased HIPK2 isoform 3 and YAP protein levels in NSCLC cells. Degradation of the YAP protein was accelerated after HIPK2 knockdown in NSCLC cells. Inhibition of HIPK2 isoform 3 decreased the mRNA expression of YAP downstream gene CTGF. The specific HIPK2 kinase inhibitor TBID decreased TEAD reporter activity, reduced cancer side populations, and inhibited tumorsphere formation of NSCLC cells. In summary, this study indicates that HIPK2 isoform 3, the main HIPK2 isoform expressed in NSCLC, promotes YAP/TEAD transcriptional activity in NSCLC cells. Our results suggest that HIPK2 isoform 3 may be a potential therapeutic target for NSCLC.

## INTRODUCTION

Homeodomain-interacting protein kinase-2 (HIPK2) can either promote or inhibit transcription depending on cellular context [1]. HIPK2 is canonically considered as a tumor suppressor because it phosphorylates p53 at Ser46 in response to ultraviolet radiation, which promotes apoptosis [2–4]. *Hipk2* was shown to be a haploinsufficient tumor suppressor gene in a radiation-induced mouse lymphoma model [5]. HIPK2 may also promote DNA damage repair pathways and protect cells against genome instability induced by radiation [6, 7]. Accumulating evidence suggests that HIPK2 may also play an oncogenic role. The sole Drosophila member of the HIPK family, Hipk, promotes Notch signaling transduction in Drosophila eye development in a kinase-dependent manner [8]. Human *HIPK2,* mapped to chromosome 7q32-q34 [9], is frequently amplified and overexpressed in pilocytic astrocytoma [10]. HIPK2 expression is significantly higher in cervical cancer than in healthy tissue [11]. HIPK2 is also significantly higher in aggressive meningiomas than in benign meningiomas [12]. *HIPK2* is positively associated with cell growth in androgen-receptor-positive prostate cancer cells [13]. In addition, HIPK2 is transcriptionally regulated by nuclear factor erythroid 2 and HIPK2 knockdown increased the sensitivity to cisplatin in non-small cell lung cancer (NSCLC) cells [14].

Hipk promotes the transcriptional activity of Yorkie (Yki), the ortholog of Yes-associated protein (YAP), to induce target gene expression and tissue growth in Drosophila [15–17]. Hipk regulates Yki in a kinase-dependent manner [15, 16]. These studies suggest that the closest human homolog of Hipk, *HIPK2*, may have the same role in mammalian cells. For instance, HIPK2 promotes abundance and activity of YAP in a kinase-dependent fashion in 293T cells [15]. Further investigation, particularly in the context of human cancer cells, is required to define the mechanism of HIPK2-YAP regulation.

HIPK2 was found to exist as several different splice variants in mammals [18]. Because the function of these splice variants may be quite different, the cellular context of these isoforms may play different roles in cancer. In this study, we focused on analysis of the main HIPK2 isoform expressed in non-small-cell lung cancer (NSCLC), which consists of adenocarcinoma and squamous cell carcinoma.

## RESULTS

### *HIPK2* gene gain and amplification in NSCLC cell lines

**Figure 1:**
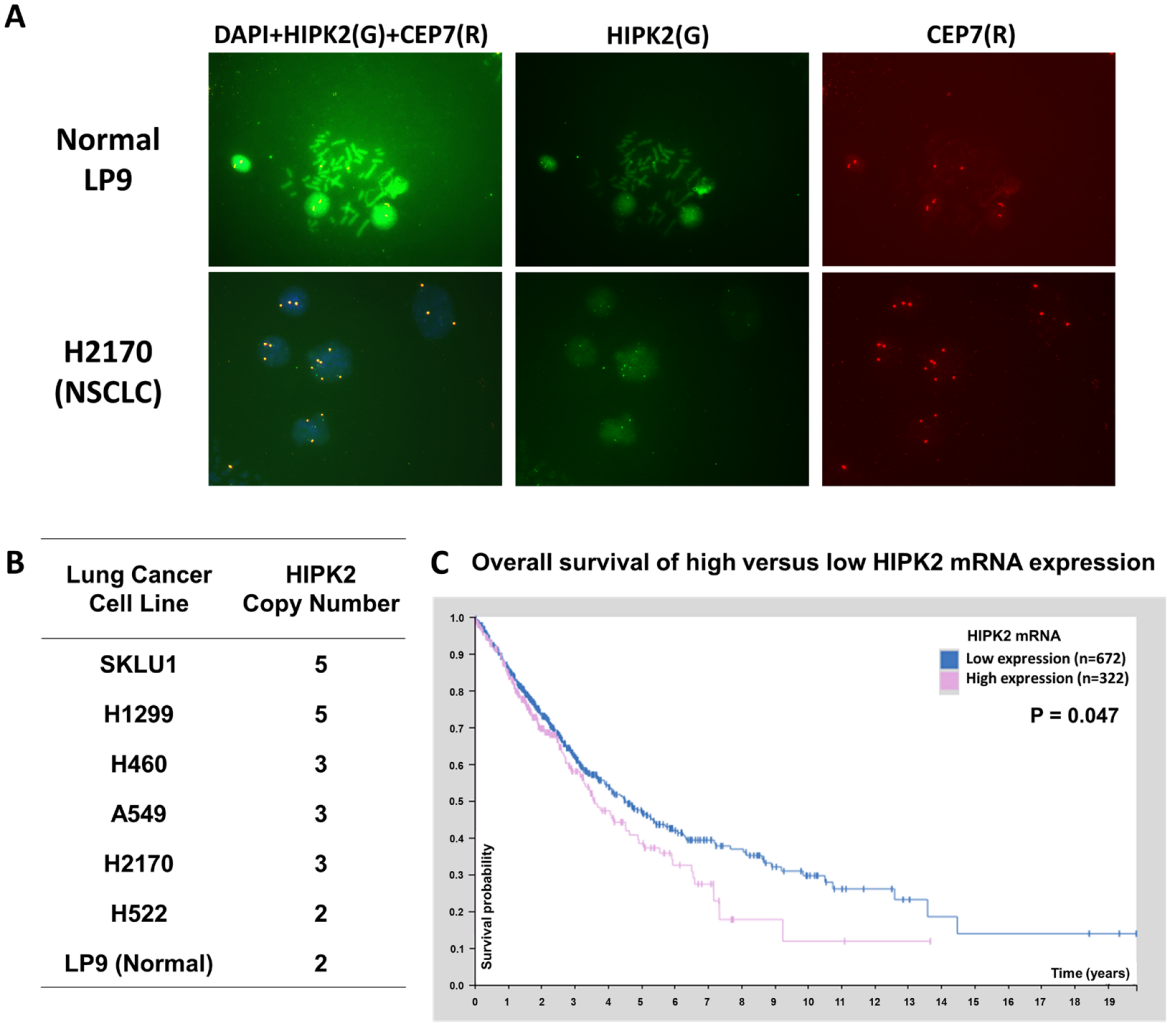
HIPK2 FISH analysis in NSCLC cell lines and HIPK2 overexpression correlates with poor survival of NSCLC patients. (**A**) FISH analysis of HIPK2 (green) and CEP7 (red) in LP9 cell line and NSCLC H2170 cell line. (**B**) Summary of FISH analysis in NSCLC cell lines. (**C**) The survival curves for NSCLC (adenocarcinoma and squamous cell carcinoma) patients with high and low HIPK2 mRNA expression in the database of the Human Protein Atlas.

We analyzed *HIPK2* copy number across the database of the Cancer Cell Line Encyclopedia [19] in cBioPortal [20] and found *HIPK2* gain and amplification in human cancer cell lines (Supplementary Table 1). According to the database, 82 (49.4%) of 166 NSCLC cell lines have gain or amplification, 61 (36.7%) have two copies and 23 (13.9%) have loss of *HIPK2* copy number. To verify *HIPK2* amplification, we analyzed 6 lung cancer cell lines (A549, H460, H522, H1299, H2170, SKLU1) and normal LP9 cells using fluorescent *in situ* hybridization (FISH). We used green fluorescein-labeled *HIPK2* FISH probe to determine the copy number of *HIPK2* and chromosome enumeration probe 7 (CEP7) to determine the copy number of chromosome 7, where the *HIPK2* gene is located (Figure 1A). We detected gain of *HIPK2* copy number (Figure 1B) in lung cancer cell lines compared to the normal LP9 cells.

### High HIPK2 mRNA expression correlates with low survival rate in NSCLC patients

The survival curve of HIPK2 mRNA expression for patients with NSCLC is shown in the database of the Human Protein Atlas (https://www.proteinatlas.org/ENSG00000064393-HIPK2/pathology/lung+cancer). The five-year survival rates are 47% for patients with low HIPK2 mRNA expression and 38% for those with high expression (*p* = 0.047) (Figure 1C).

### N-terminal HIPK2 is frequently overexpressed in NSCLC tissues

**Figure 2:**
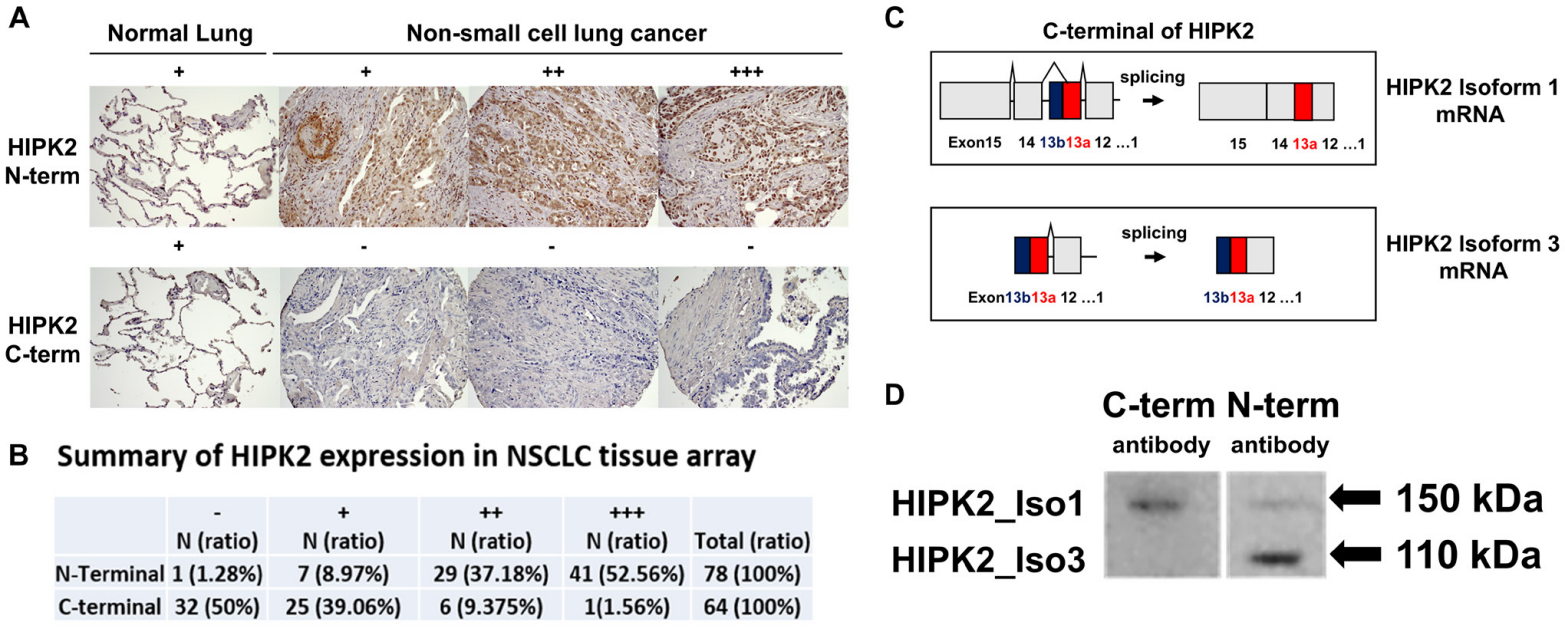
Immunohistochemistry of HIPK2 in NSCLC tissue array and detection of two different HIPK2 isoforms (110 kDa and 150 kDa) in NSCLC cell line H1975. (**A**) Normal lung expresses N-terminal HIPK2 and C-terminal HIPK2 at weak intensity level. Human NSCLC expresses N-terminal HIPK2 at weak (+), moderate (++), and strong (+++) intensity level. Human NSCLC expresses C-terminal HIPK2 at weak (+) intensity level. Images were taken under 10× or 20× objective lens. (**B**) Summary of HIPK2 expression in NSCLC tissue array. (**C**) Diagram of HIPK2 isoform 1 mRNA and HIPK2 isoform 3 mRNA. HIPK2 isoform 3 contains exon 13b and no exons 14–15. (**D**) The N-terminal antibody detects HIPK2 isoform 1 (150 kDa) and HIPK2 isoform 3 (110 kDa) on Western blot in the human NSCLC cell line H1975. The C-terminal antibody detects only HIPK2 isoform 1.

Immunohistochemistry (IHC) analysis of N-terminal HIPK2 expression in human NSCLC showed negative staining (–) in 1.3% of 78 tumor samples, weak staining (+) in 9.0%, moderate staining (++) in 37.2%, and strong staining (+++) in 52.6% (Figure 2A, Supplementary Table 2). IHC analysis of C-terminal HIPK2 expression showed negative staining (–) in 50% of 64 tumor samples, weak staining (+) in 39.1%, moderate staining (++) in 9.4%, and strong staining (+++) in 1.6% (Figure 2A and 2B). IHC analysis of N-terminal HIPK2 expression in human normal lung tissues showed negative staining (–) in 21.1% of 90 tumor samples, weak staining (+) in 78.9%, moderate staining (++) in 0%, and strong staining (+++) in 0% (Supplementary Table 3). The anti-HIPK2 antibodies are N-terminal specific (ab28507; abcam) and C-terminal specific (#5091S; Cell Signaling, Beverly, MA, USA). These findings suggest there is an oncogenic HIPK2 splice variant with a C-terminal truncation.

### Identification of novel isoform 3 of *HIPK2* in NSCLC

There are two validated isoforms in the National Center for Biotechnology Information (NCBI), HIPK2 isoform 1 (NP_073577.3, NM_022740.5) and isoform 2 (NP_001106710.1, NM_001113239.3) contain 15 exons including exon 13a [21, 22]. An alternatively spliced HIPK2 (AF207702.1) which contains exon 13a and 13b but not exons 12, 14 and 15 is also listed in NCBI. We designed cloning primers based on the sequences of the alternatively spliced HIPK2 (AF207702.1). The open reading frame was amplified using a forward primer GAATTCATGGCCCCCGTGTACGAAGGT (underline indicates EcoRI site) and a reverse primer CTCGAGCGAGCTCCC ATACAGCAACAT (underline indicates XhoI site) with HepG2 cDNA (Sigma-Aldrich, St. Louis, MO, USA). The PCR fragment was inserted into pCR2.1-TOPO vector (Invitrogen Life Technologies, Waltham, MA, USA) and a novel isoform was found, which we named HIPK2 isoform 3. This isoform contains exons 1-13a and 13b (Figure 2C). When we subcloned HIPK2 isoform 3 into pcDNA3.1/myc-His A plasmid (Invitrogen Life Technologies) we detected the HIPK2 isoform 3 protein at 110 kDa by western blot (Supplementary Figure 1). We also purchased HIPK2 (Myc-DDK-tagged) isoform 1 (NM_022740) Human cDNA ORF Clone (ORIGENE, catalog no. RC220278) and detected the HIPK2 isoform 1 protein at 150 kDa by western blot (Supplementary Figure 2). See Supplementary Materials about HIPK2 isoform 3 sequence.

### HIPK2 isoform 3 is the main HIPK2 isoform expressed in NSCLC cell lines and tissues

HIPK2 isoform 3 was detected in NSCLC H1975, A549, H1299, H460, H2030, and H2170 cell lines (Supplementary Figure 3). HIPK2 isoform 1 was only detectable at a much lower level in NSCLC H1975 cells (Figure 2D). The antibody against the HIPK2 N-terminal (ab28507; abcam) was used to detect both HIPK2 isoform 1 and 3. The antibody against the HIPK2 C-terminal (#5091S; Cell Signaling, Beverly, MA) can only detect HIPK2 isoform 1 (Figure 2D). Using N-terminal antibody, we analyzed HIPK2 in 24 matched NSCLC tissues and normal lung tissues with western blot. HIPK2 isoform 3 (110 kDa) was the only isoform detected in these tissues—in 12.5% (3/24) of normal tissues and 54.2% (13/24) of tumor tissues. HIPK2 isoform 1 (150 kDa) was not detectable in these tissues (Supplementary Figure 4).

### *HIPK2* isoform 3 inhibition decreased YAP protein level in NSCLC cells

**Figure 3:**
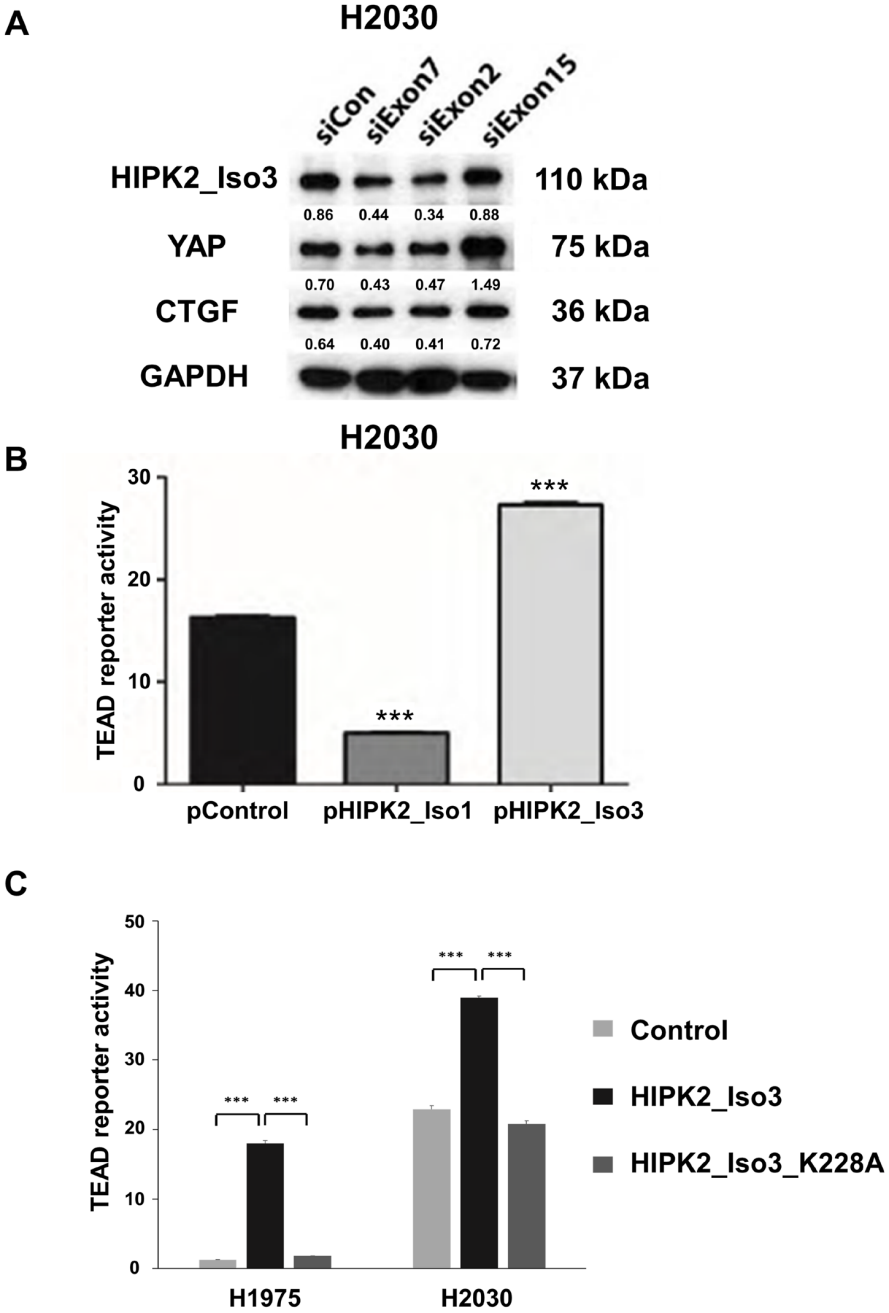
Forced overexpression of the HIPK2 Isoform 3 promotes TEAD reporter activity and two HIPK2 siRNAs decreased YAP protein levels in NSCLC cells. (**A**) Western blot analysis of protein levels of HIPK2 isoform 3, YAP and CTGF in H2030 cell lines after siRNA knockdown of HIPK2 at exon 7, exon 2, or exon 15 (**B**) TEAD reporter activity was significantly lower after forced-expression of HIPK2 isoform 1 in H2030 cell lines (*p* < 0.001, *t*-test). TEAD reporter activity was significantly higher after forced-expression of HIPK2 isoform 3 (Iso3) (*p* < 0.001, *t*-test). (**C**) TEAD reporter activity in H2030 and H1975 cell lines was significantly lower after forced-expression of HIPK2 isoform 3 K228A mutation than after forced-expression of HIPK2 isoform 3 (*p* < 0.001, *t*-test). Error bars indicate standard deviations; ^***^ *p* ≤ .001.

In human NSCLC H2030 cells, the siRNAs targeting exons 7 and 2 of *HIPK2* reduced the protein level of HIPK2 isoform 3 (110 kDa) and decreased YAP protein and its downstream CTGF protein levels. The siRNA that targets exon 15 of *HIPK2* did not reduce the protein level of HIPK2 isoform 3 and increased the YAP protein level (Figure 3A). Accordingly, HIPK2 isoform 3 may have a role in controlling the Hippo-YAP pathway.

### Forced-expression of HIPK2 isoform 3 increased TEAD reporter activity in NSCLC cells

In H2030 cells, forced-expression of *HIPK2* isoform 3 significantly increased Hippo reporter activity (*p* < 0.001), whereas forced-expression of *HIPK2* isoform 1 significantly reduced TEAD reporter activity (*p* < 0.001) (Figure 3B). To understand the mechanism of HIPK2 isoform 3 in promoting TEAD reporter activity, we performed mutagenesis of HIPK2 isoform 3 at Lys228 (K228) to generate mutated HIPK2 isoform 3 (K228A). We transfected lung cancer H2030 and H1975 cells with the plasmid construct and measured TEAD reporter activity in the cells. Forced *HIPK2* isoform 3 expression showed increased TEAD reporter activity in these cells compared to control cells (*p* < 0.001) (Figure 3C). Expressing the mutation construct K228A decreased TEAD reporter activity compared to wild-type cells (*p* < 0.001) (Figure 3C). Our results indicate that Lys228 (K228) is the kinase active site of HIPK2 isoform 3 in regulating TEAD reporter activity.

### HIPK2 inhibition decreased YAP protein stability and decreased mRNA expression of downstream gene CTGF in NSCLC cells

**Figure 4:**
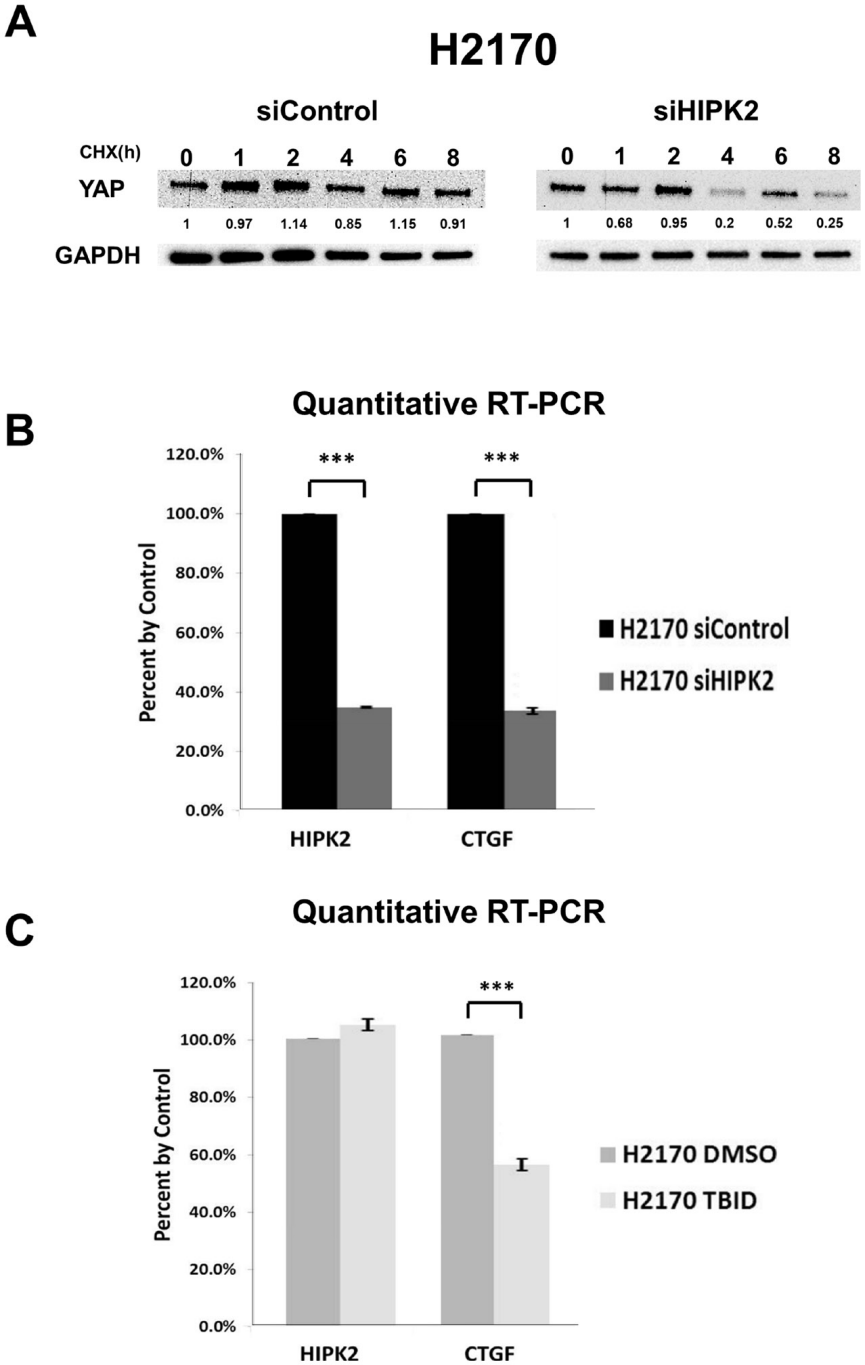
HIPK2 inhibition decreased the mRNA expression of YAP downstream gene CTGF. (**A**) YAP protein degradation assay in human NSCLC H2170 cell line after siRNA knockdown. (**B**) Quantitative RT-PCR of *HIPK2* and *CTGF* after siHIPK2 in H2170 cell line (*p* < 0.001, *t*-test). (**C**) Quantitative RT-PCR of *HIPK2* and *CTGF* after TBID treatment in H2170 cell line (*p* < 0.001, *t*-test). Error bars indicate standard deviations; ^***^ *p* ≤ .001.

The HIPK2 siRNA (siExon2) decreased YAP protein stability in H2170 lung cancer cells (Figure 4A). YAP in H2170 cells knocked down with siHIPK2 was reduced by about a quarter of the control YAP after 4 hours. These experiments were done in three times. Quantitative RT-PCR analysis showed that *HIPK2* siRNA (siExon2) significantly knocked down *HIPK2* mRNA expression and YAP/TEAD downstream gene *CTGF* mRNA expression in H2170 cells (*p* < 0.001) (Figure 4B). The 4,5,6,7-tetrabromo-2-(1H-imidazol-2-yl)isoindoline-1,3-dione (TBID) is a selective and cell-permeable HIPK2 inhibitor, and structure modeling predicts the interaction of TBID with the Lys228 (K228) kinase active site [23]. Treatment with 10 μM TBID significantly decreased *CTGF* mRNA expression but had no effect on HIPK2 expression (Figure 4C). The dose was based on the IC_50_ (5.8 μM, 72 hrs) in H2170 cells.

### TBID decreased cancer stem cells and TEAD reporter activity in NSCLC cells

**Figure 5:**
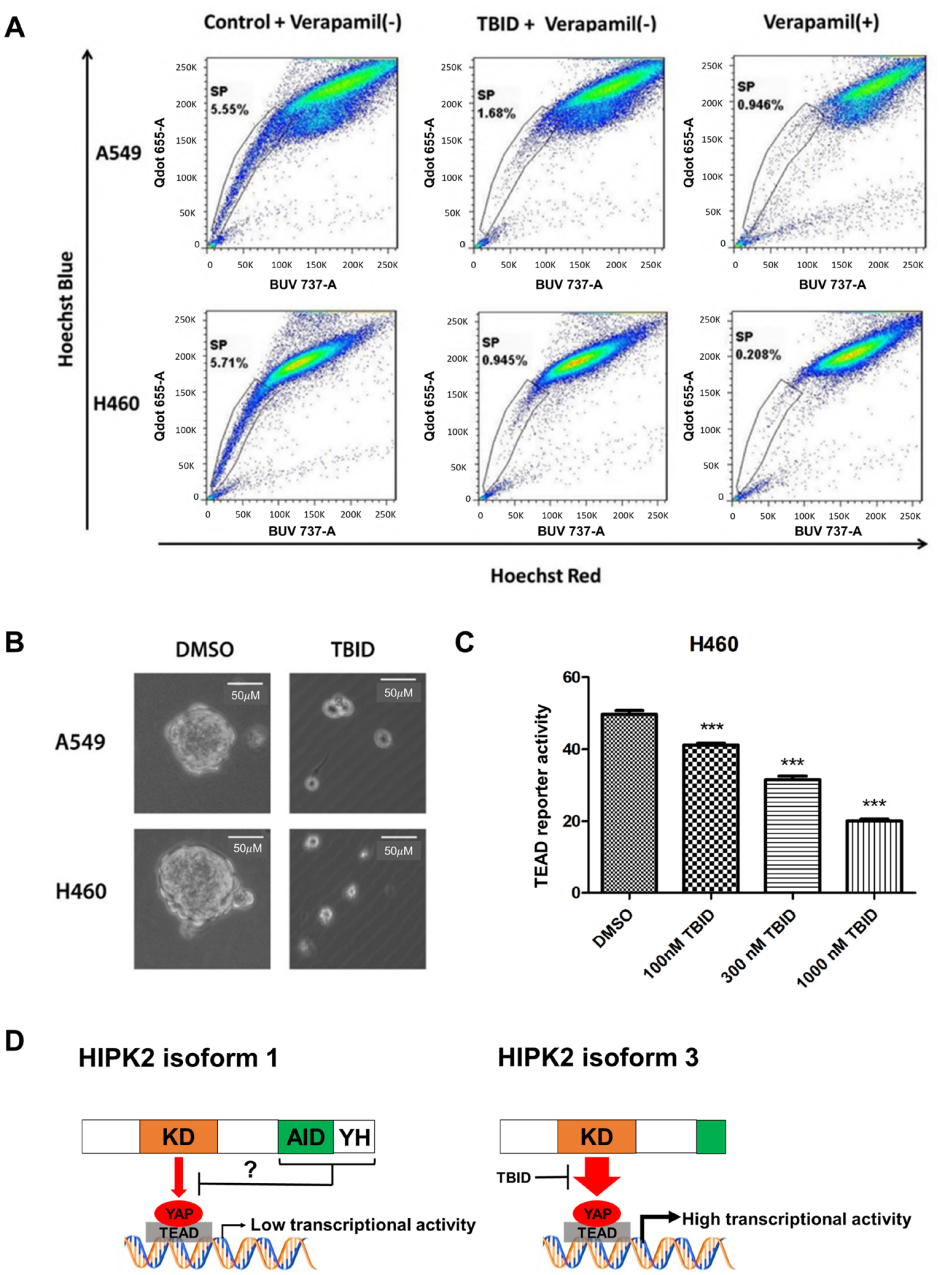
Analysis of side population, sphere formation and TEAD reporter activity after TBID treatment in NSCLC cell lines. (**A**) Reduction of side population of human NSCLC A549 and H460 cell lines after TBID treatment. (**B**) Inhibition of secondary tumorsphere formation by TBID treatment in NSCLC cell lines. (**C**) TEAD reporter activity decreased in a dose-dependent manner in H460 cell line (*p* < 0.001, *t*-test). Error bars indicate standard deviations; ^***^ *p* ≤ .001. (**D**) Schematic diagram of the potential differences in YAP/TEAD regulation between HIPK2 isoform 1 and isoform 3.

To test if TBID can reduce the population of cancer stem cells, we performed side population and 3D tumorsphere analysis in NSCLC cells. In our previous studies, NSCLC cell lines A549 and H460 showed side population characteristics [24, 25]. After treatment with 10 μM TBID **(**based on the IC_50_ (6.7 μM, 72 hrs) in H460 cells and (10.8 μM, 72 hrs) in A549 cells), the side population decreased from 5.5% to 1.7% in A549 cells, and from 5.7% to 0.95% in H460 cells (Figure 5A). TBID reduced tumorsphere size and number in A549 and H460 cells (Figure 5B). It also significantly decreased TEAD reporter activity in a dose-dependent manner in H460 cell lines (*p* < 0.001) (Figure 5C).

## DISCUSSION

In this study, we detected increased *HIPK2* DNA copy number in five of the six NSCLC cell lines analyzed. The N-terminal HIPK2 was expressed in 89% of NSCLC tissues and the C-terminal HIPK2 was only expressed in 11% of NSCLC tissues. The novel HIPK2 isoform (110 kDa) was detected as the dominant HIPK2 isoform expressed in NSCLC cell lines (6/6) and tissues (13/24), and the HIPK2 isoform 1 (150 kDa) was only detected at lower levels in one of the six NSCLC cell line (H1975) and not detected in NSCLC tissues (0/24) (Supplementary Figures 2, 3). These results suggest the novel HIPK2 isoform may play an oncogenic role in NSCLC. Thus, we cloned this novel HIPK2 isoform and investigated its relationship with YAP.

Since there were currently two validated isoforms in the NCBI, we named this new HIPK2 isoform as HIPK2 isoform 3. It contains 13 exons: 1-12, 13a and 13b. Compared to HIPK2 isoform 1, HIPK2 isoform 3 has an extra exon 13b and lacks exons 14 and 15. The protein product of the cloned HIPK2 isoform 3 was detected at 110 kDa by western blotting. Furthermore, the siHIPK2s (exon 2, 7) decreased the110 kDa bands in H2030 cell lines. We found that forced overexpression of the HIPK2 Isoform 3 promotes TEAD reporter activity in H1975 and H2030 cell lines. Two siRNAs targeting HIPK2 decreased HIPK2 Isoform 3 and YAP protein levels in NSCLC cells. In previous work, we used a YAP protein stability assay with extracellular signal-regulated kinase (ERK) inhibition in the squamous lung cancer cell line H2170 [26]. Degradation of YAP protein was accelerated after HIPK2 knockdown in H2170 cells. The protein level of YAP/TEAD downstream gene CTGF was decreased by the two siHIPK2s. These results suggest that HIPK2 isoform 3 promotes YAP/TEAD transcriptional activity and may play an oncogenic role in NSCLC.

HIPK2 isoform 1 has an auto-inhibitory domain (AID) and a YH domain [27, 28]. HIPK2 isoform 1 kinase activity may be inhibited by the AID [27, 29, 30] and/or YH domain [31]. Deletions of the AID and YH domains abolished the ability of HIPK2 isoform 1 to suppress the Wnt/ß-catenin pathway, suggesting that the integrity of the C-terminal region of HIPK2 isoform 1 is important for the recruitment of transcriptional corepressors [31]. These findings are similar to our findings that HIPK2 isoform 3 (without AID and YH domains) increases TEAD reporter activity and siHIPK2 specific to isoform 1 increases YAP protein, whereas overexpression of HIPK2 isoform 1 suppresses TEAD reporter activity in H2030 cells. Our data suggest that the AID and YH domains of HIPK2 isoform 1 negatively regulate YAP/TEAD activity (Figure 5D).

Our findings using HIPK2 isoform 1 plasmid are the opposite of those of Poon et al. in the 293T cell line [16]. Several explanations for this difference are possible. One is that context-dependent function of HIPK2 isoform 1 in Wnt signaling has been reported [1, 32, 33], and a positive or negative role of HIPK2 on Wnt signaling depends on the availability of particular T-cell factor (TCF) proteins in the cell. HIPK2 isoform 1 would inhibit the pathway upon phosphorylation of an activator type TCF, but would activate it upon phosphorylation of a repressor type TCF. A similar context-dependent function of HIPK2 isoform 1 may exist in the Hippo signaling pathway. In addition, the AID of HIPK2 isoform 1 inhibit potential YAP phosphorylation by KD of HIPK2 and affect its stability depending on the relative cellular level of HIPK2 isoform 3 [15, 17] (Figure 5D). For instance, forced expression of HIPK2 isoform 1 may induce a certain level of TEAD reporter activity in the absence of HIPK2 isoform 3. Conversely, forced expression of HIPK2 isoform 1 may reduce the relative high level of TEAD reporter activity in the presence of high level HIPK2 isoform 3.

Recently, we discovered the first crystal structure of HIPK2 and found that its catalytic lysine (K228) is critical for binding HIPK2 to a small molecule HIPK2 inhibitor [34]. TBID, the first and selective HIPK2 inhibitor, is also predicted to interact with HIPK2 at K228 [23]. In this study, HIPK2 isoform 3 site-mutagenesis K228A failed to increase TEAD reporter activity, suggesting that the positive YAP regulation of isoform 3 is dependent on its kinase activity. TBID treatment reduced TEAD reporter activity of NSCLC cells in a dose-dependent manner. In addition, TBID reduced cancer stem cell populations and inhibited tumorsphere formation of NSCLC cells. Thus, these data suggest that K228 in the kinase domain is a critical site for HIPK2 oncogenic function and future development of HIPK2-targeting therapy such as small molecules.

In summary, this study suggests that HIPK2 isoform 3 promotes YAP/TEAD transcriptional activity and it may play an oncogenic role in NSCLC. Our results also suggest that HIPK2 isoform 3 may be a potential therapeutic target for NSCLC.

## MATERIALS AND METHODS

### Tissue samples and immunocytochemistry

Tissue microarray sections contained fresh lung tumor and adjacent normal lung tissues from patients with NSCLC who were undergoing surgical resection of the primary tumor. Primary human NSCLC samples from 115 patients were fixed in formalin and embedded in paraffin in 4-μm tissue microarray sections. In 10 of these patients, a small amount of normal lung tissue had been obtained simultaneously to serve as controls. All human tissue samples were obtained and analyzed in accordance with procedures approved by the institutional review board of the University of California, San Francisco (IRB H8714-22942-01). Tissue microarrays were stained with hematoxylin and eosin for general morphology analysis. For IHC analysis, endogenous peroxidase was quenched for 15 min. at room temperature with 3% H2O2 in methanol in each lung section. Sections were blocked with 4% normal goat serum in PBS with 0.2% Triton for 2 hrs at room temperature before incubation overnight at 4°C with the properly diluted antibodies. The N-terminal specific anti-HIPK2 antibody (ab28507; abcam) and the C-terminal specific anti-HIPK2 antibody (#5091S; Cell Signaling, Beverly, MA, USA) were used. Three independent researchers blindly scored positivity, and the data represent the samples that were scored positive by all three individuals. The following scoring system was used: −, no stain; +, weak staining (≤ 10% stained cellularity considered as positive); ++, moderate staining (> 10% but ≤ 30% stained cellularity considered as positive); +++, strong staining (> 30% but ≤ 50% stained cellularity considered as positive). All scoring was done under an objective lens (×20) with a Zeiss Axioscop 2 microscope (Carl Zeiss, Jena, Germany) and photomicrographs were obtained with a Carl Zeiss AxioCam MrC5 camera under a 20× or 40× objective lens.

### Cell culture

Human NSCLC cell lines (A549, H460, H1299, H2170, H1975, H2170, H2030, SKLU1), were purchased from American Type Culture Collection (Manassas, VA, USA). Normal LP-9 cell line was purchased from the Cell Culture Core Facility at Harvard University (Boston, MA, USA). All human cancer cell lines were cultured in RPMI 1640 medium supplied with 10% fetal bovine serum (FBS) and 1% penicillin-streptomycin. Normal LP-9 cells were cultured using Ham’s F12 medium/Medium 199 (1:1 mixture) with 15% FBS, 2 mM L-glutamine, 1.7 nM epidermal growth factor and 1100 nM hydrocortisone. All the cells were cultured at 37°C and 5% CO_2_.

### Fluorescence *in situ* hybridization analysis

Fluorescence *in situ* hybridization analysis was performed on metaphase slides of 6 NSCLC cell lines (A549, H460, H522, H1299, H2170, SKLU1) and the human mesothelial LP-9 cell line. The slides were probed with *HIPK2* FISH probe (*HIPK2*-20-GR, empire genomics) and Vysis CEP 7 (D7Z1) SpectrumOrange Probe (06J36-007, Abbott). Genomic copy numbers of *HIPK2* were determined by digital image microscopy after FISH.

### RNA interference

Cells were seeded in a 6-well plate with fresh media without antibiotics 24 hrs before transfection, with a target of 30–50% confluency at the time of transfection. HIPK2 Exon2 siRNA (ID: s57556 catalog No: 4392420), HIPK2 Exon7 siRNA (ID: hss120797 catalog No: 1299001), HIPK2 Exon15 siRNA (ID: 109493 catalog No: AM16708) and control siRNA were purchased from Thermo Scientific (Waltham, MA, USA). Cells were transfected with 100 nmol/l of siRNA using Lipofectamine RNAiMAX (Invitrogen, Carlsbad, CA, USA) according to the manufacturer’s protocol. After siRNA transfection, the plates were incubated for 48 hrs at 37°C before further analysis.

### Semi-quantitative RT–PCR

Total RNA from the various cell lines was isolated using the RNeasy extraction method (Qiagen, Valencia, CA, USA). First-strand cDNA was synthesized from total RNA by iScript cDNA synthesis (Bio-Rad, Hercules, CA, USA) according to the manufacturer’s instructions. Taqman RT–PCR analysis was performed on cDNA in a 384-well plate, using a Prism 7900HT Real-Time PCR System (Applied Biosystems, Foster City, CA, USA). Primers and Taqman probes for human *HIPK2* and human GUSB were purchased from Applied Biosystems. The expression of target gene in each sample was assayed in triplicate and normalized to human GUSB for mRNA expression analysis. Decreased transcriptional levels of *HIPK2* were calculated by dividing the transcriptional levels measured in *HIPK2* siRNA samples from those in the control samples.

### Western blotting

NSCLC cell lines were seeded in a 6-well plate as 500,000 cells/well and cultured at 37°C supplied with 5% CO2 without antibiotics for 24 hrs. Cells were then transfected with *HIPK2* siRNA (siHIPK2) or control siRNA (siCtrl). Cell lysates were then immunoblotted using the N-terminal specific anti-HIPK2 antibody (ab28507; abcam; Immunogen: a peptide corresponding to residues 37-86 of human HIPK2) and the C-terminal specific anti-HIPK2 antibody (#5091S; Cell Signaling, Beverly, MA; Immunogen: a peptide corresponding to residues surrounding Gln1045 of human HIPK2)., anti-YAP (#4912S; Cell signaling), anti-GAPDH (#2188S; Cell signaling) or anti-β-actin (#4970; Cell Signaling) antibodies. NIH ImageJ was used to quantify the intensity of western blot bands, and the relative protein expression levels were calculated by normalizing with GAPDH protein levels.

### Luciferase reporter assays

The TEAD binding site was originally reported as the GTIIC site (ACATTCCA) in Hela cell [35]. The 8xGTIIC-luciferase was a gift from Stefano Piccolo (Addgene plasmid # 34615; http://n2t.net/addgene:34615;RRID:Addgene_34615). The 8×GTIIC plasmid was transfected in NSCLC cell lines. *HIPK2* knockdown assays require co-transfection of *HIPK2* siRNA or control siRNA with 8xGTIIC plasmid into the cells in a 24-well plate. The Renilla luciferase pRL-TK plasmid (Promega, Madison, WI, USA), whose expression is driven by the housekeeping thymidine kinase gene promoter, was co-transfected to normalize for transfection efficiency. All transfection experiments were performed using Lipofectamine2000 (Invitrogen) in accordance with the manufacturer’s instructions. After 48 hrs cells were lysed and luciferase assays were performed following the manufacturer’s instructions. Results are expressed as fold induction, which is the ratio of luciferase activity induced in 8xGTIIC-transfected cells relative to basal luciferase activity in control transfected cells. All experiments were performed in triplicate. Means and standard deviations were calculated.

### Protein degradation assay

Human NSCLC H2170 cells with *HIPK2* knockdown by siRNA were treated with 100 μg/ml cycloheximide, the inhibitor of protein synthesis, and harvested at the time points of 0, 1, 2, 4, 6 and 8 hrs. Total proteins were extracted and expression of YAP was analyzed by western blotting.

### Tumorsphere cultures

Tumorspheres were cultured in Stem Cell media (Gibco, Grand Island, NY, USA) following the manufacturer’s instructions. When spheres reached 150–200 lM, they were broken up by trituration with a 26-gauge needle 10 times. After seven days, the secondary sphere formation was assayed with (TBID 10 μM) or without drug inhibitor present. The spheres with diameter larger than 60 μm in 9 fields were counted.

### Side population assay

The compound efflux ability of cancer stem cells comes from the increased expression of ATP-binding cassette (ABC) transporters within the cell membrane and serves as the basis of an important flow-cytometry-based cell-sorting assay called the side population (SP) assay [36, 37]. Side population staining was performed as described [38]. Briefly, 1 × 10^6^/ml cells were re-suspended in RPMI 1640 containing 2% fetal bovine serum and labeled with H33342 (Sigma-Aldrich GmbH, Steinheim, Germany) at a concentration of 5.0 μg/ml for 60 min in a 37°C water bath, either alone or with 100 μM verapamil hydrochloride (Sigma-Aldrich GmbH) and 10 μM TBID (Pharmazeutische Chemie, Germany) for 72 hours. Tubes were gently inverted every 20 min and then centrifuged at 400 × g for 5 min at 4°C. The pellets were resuspended in cold PBS containing 2 μg/ml propidium iodide. The resuspended cells were passed through a 40-μm mesh filter and maintained at 4°C in the dark until flow cytometry analysis was done on a BD FACS Aria II. Hoechst dye was excited at 355 nm (32), and fluorescence was measured at two wavelengths using a 450/50-nm (blue) band-pass filter and a 670/30-nm (33) long-pass edge filter. Cells were displayed on dot-plots gated on live cells, PI negative, and viewed in a Hoechst Blue versus Hoechst Red dot-plot to visualize the side population. As a positive control, ABC transporter inhibitor verapamil, at the bottom-left corner in the flow cytometry plots, disappeared or faded out. The position where the tail disappeared was used as a control to gate the area of side population cells.

### Statistical analysis

The data shown represent mean ± SD. The chi-square independence test was used to compare IHC results between the staining intensity of HIPK2 and YAP in the same NSCLC tumors or cell lines. Student’s *t*-test was used to compare gene expression results and luciferase reporter activities between experimental and control groups. *p* values < 0.05 were considered significant.

## SUPPLEMENTARY MATERIALS





## Abbreviations

HIPK2: Homeodomain-interacting protein kinase-2
Yki: Yorkie
YAP: Yes-associated protein
NSCLC: non-small-cell lung cancer
TBID: 4,5,6,7-tetrabromo-2-(1H-imidazol-2-yl)isoindoline-1,3-dione
NRF2: nuclear factor erythroid 2
FISH: fluorescent *in situ* hybridization
CEP7: chromosome enumeration probe 7
IHC: Immunohistochemistry
FBS: fetal bovine serum
AID: auto-inhibitory domain
YH: tyrosine/histidine-rich
TCF: T-cell factor
(ABC) transporters: ATP-binding cassette
