# Supplementary material for: A novel isoform of Homeodomain-interacting protein kinase-2 promotes YAP/TEAD transcriptional activity in NSCLC cells
Source: Oncotarget. 2021 Feb 2;12(3):173–84. doi: 10.18632/oncotarget.27871 (PMC7869571; doi:10.18632/oncotarget.27871)
Supplement: Supplementary file 1 [file oncotarget-12-173-s001.pdf]

A novel isoform of Homeodomain-interacting protein kinase-2 promotes YAP/TEAD transcriptional activity in NSCLC cells

SUPPLEMENTARY MATERIALS

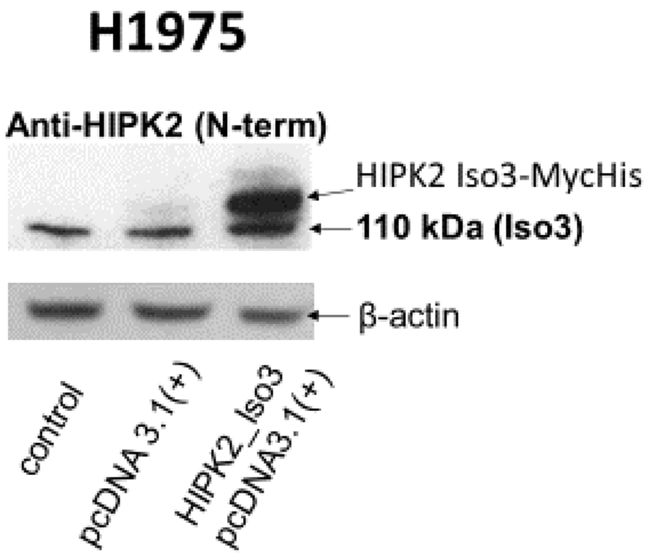

Supplementary Figure 1: HIPK2 Isoform 3 validation by western blotting. HIPK2 Isoform 3 detected 110 kDa with MycHis Tag.

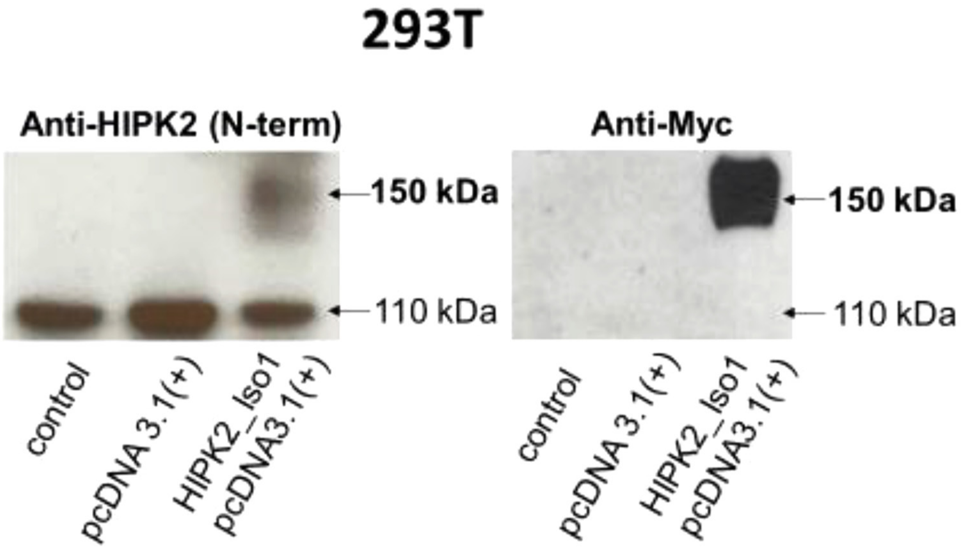

Supplementary Figure 2: HIPK2 Isoform 1 validation by western blotting. HIPK2 isoform 1 detected 150 kDa with Myc Tag.

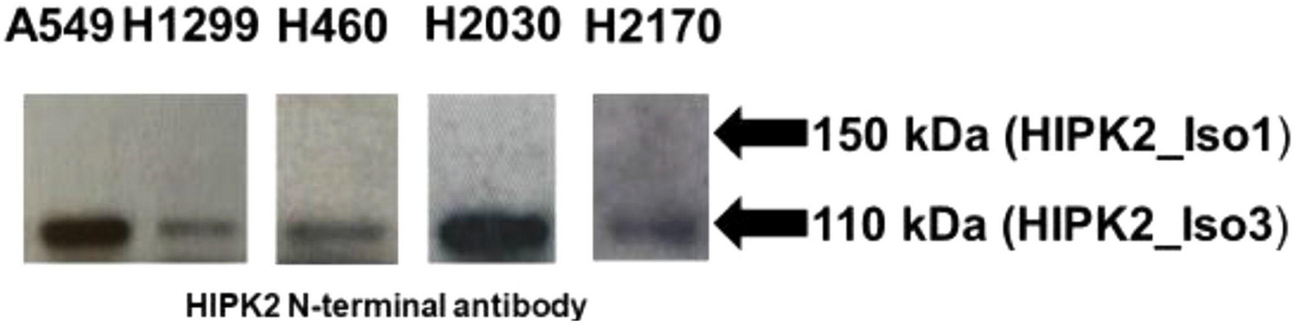

Supplementary Figure 3: HIPK2 isoform 3 (110 kDa) only detected with HIPK2 N-terminal antibody in cancer cell lines.

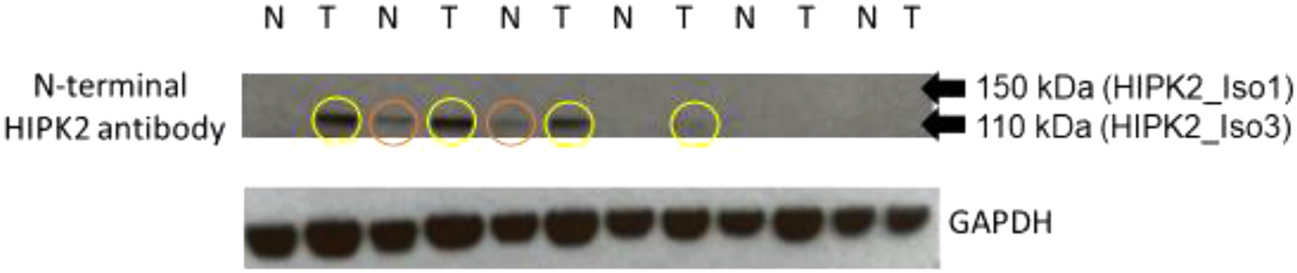

Supplementary Figure 4: HIPK2 isoform 3 in Normal Tissue (N) and Tumor Tissue (T). We only show 6 of the 24 tissue samples. HIPK2 isoform 3 (110 kDa) were detected 3/24 in Normal Tissue and 13/24 in Tumor Tissue. HIPK2 isoform 1(150 kDa) was not detected.

Supplementary Table 1: HIPK2 copy number in many human cancer cell lines

HIPK2 copy number	2	1	0	–1	–2	total
Lung	4	78	61	22	1	166
Haematopoietc and lymphoid tissue	5	54	87	14	3	163
Large intestin	2	27	23	2	0	54
Skin	5	35	10	0	1	51
Breast	2	24	16	9	0	51
Central Nervous system	1	29	8	6	0	44

We analyzed *HIPK2* copy number across the database of cBioPortal [27] for Cancer Genomics and found *HIPK2* amplification or gain in many human cancer cell lines, including haematopoietic and lymphoid tissue (36.2%, n = 163), central nervous system (68.2%, n = 44), skin (78.4%, n = 51), breast (54.9%, n = 51), and large intestine (55.6%, n = 54).

Of 166 lung cancer cell lines, 82(49.4%) have amplification or gain, 61(36.7%) have diploid, and 23 (13.9%) have loss of *HIPK2* copy number.

cBioPortal dataset

These levels are derived from copy-number analysis algorithms like GISTIC or RAE, and indicate the copy-number level per gene:

- –2 or Deep Deletion indicates a deep loss, possibly a homozygous deletion.
- –1 or Shallow Deletion indicates a shallow loss, possibley a heterozygous deletion.
- 0 is diploid.
- 1 or Gain indicates a low-level gain (a few additional copies, often broad).
- 2 or Amplification indicate a high-level amplification (more copies, often focal).

Supplementary Table 2: Summary of IHC analysis of N-terminal and C-terminal HIPK2 in NSCLC TMA

Sample No.	N-terminal	C-terminal	Sample No.	N-terminal	C-terminal
T01	NA	NA	T46	+++	NA
T02	+++	-	T47	+++	+
T03	+	NA	T48	++	-
T04	++	+	T49	+++	+
T05	++	-	T50	++	NA
T06	+++	+++	T51	++	++
T07	++	-	T52	+++	NA
T08	++	-	T53	+++	NA
T09	+++	-	T54	+++	NA
T10	+++	-	T55	+	+
T11	+	+	T56	+	+
T12	++	+	T57	++	+
T13	+++	-	T58	NA	NA
T14	+++	-	T59	++	+
T15	++	+	T60	+++	-
T16	++	-	T61	+++	-
T17	+++	-	T62	NA	NA
T18	++	+	T63	++	-
T19	+++	+	T64	+++	++
T20	++	-	T65	+	++
T21	NA	NA	T66	+++	++
T22	+++	-	T67	+++	-
T23	++	-	T68	++	-
T24			T69	+++	-
T25	+++	+	T70	+++	NA
T26	++	-	T71	+++	+
T27	++	-	T73	+++	-
T28	+++	+	T74	++	+
T29	++	-	T75	+++	NA
T30	+	+	T76	++	NA
T31	+++	+	T78	+++	-
T32	++	-	T79	++	-
T34	+++	+	T80	++	++
T35	++	++	T82	+++	+
T36	+++	NA	T83	++	+
T37	-	-	T84	+	-
T38	+++	+	T86	+++	NA
T39	NA	NA	T87	+++	-
T40	+++	NA	T88	++	+
T42	+++	NA	T89	+++	NA
T43	NA	NA	T90	++	+
T44	+++	-	T91	+++	-
T45	+++	+			

– = no stain; + = weak stain; ++ = moderate stain; +++ = strong stain; NA = missing sample.

Supplementary Table 3: Summary of N-terminal HIPK2 expression in normal lung TMA

	–	+	++	+++	
	**N (ratio)**	**N (ratio)**	**N (ratio)**	**N (ratio)**	**Total (ratio)**
N-terminal	19 (21.1%)	71 (78.9%)	0	0	90 (100%)

– = no strain; + = weak stain; ++ = moderate stain; +++ = strong stain.
