# Supplementary material for: A novel isoform of Homeodomain-interacting protein kinase-2 promotes YAP/TEAD transcriptional activity in NSCLC cells
Source: Oncotarget. 2021 Feb 2;12(3):173–84. doi: 10.18632/oncotarget.27871 (PMC7869571; doi:10.18632/oncotarget.27871)
Supplement: Supplementary file 2 [file oncotarget-12-173-s002.docx]

**Supplementary Materials**

**1. HIPK2 isoforms:**

- **HIPK2 Isoform 1:** [NP_073577.3](https://www.ncbi.nlm.nih.gov/protein/NP_073577.3); NM_022740.5; Length: 1198 aa.
- **HIPK2 Isoform 2:** [NP_001106710.1](https://www.ncbi.nlm.nih.gov/protein/NP_001106710.1); NM_001113239.3; Length: 1171 aa.
- **HIPK2 Isoform 3:** Length: 1018 aa.

# HIPK2 alternatively spliced: AF207702.1; Length: 911 aa.

# 2. HIPK2 isoform 3 sequence (Length: 1018 aa)

| 1 1 61 21 121 41 181 61 241 81 301 101 361 121 421 141 481 161 541 181 601 201 661 221 721 241 781 261 841 281 901 301 961 321 1021 341 1081 361 1141 381 1201 401 1261 421 1321 441 1381 461 1441 481 1501 501 1561 521 1621 541 1681 561 1741 581 1801 601 1861 621 1921 641 1981 661 2041 681 2101 701 2161 721 2221 741 2281 761 2341 781 2401 801 2461 821 2521 841 2581 861 2641 881 2701 901 2761 921 2821 941 2881 961 2941 981 3001 1001 | ATGGCCCCCGTGTAC GAAGGTATGGCCTCA CATGTGCAAGTTTTC TCCCCTCACACCCTT  M  A  P  V  Y   E  G  M  A  S   H  V  Q  V  F   S  P  H  T  L  CAATCAAGTGCCTTC TGTAGTGTGAAGAAA CTGAAAATAGAGCCG AGTTCCAACTGGGAC  Q  S  S  A  F   C  S  V  K  K   L  K  I  E  P   S  S  N  W  D  ATGACTGGGTACGGC TCCCACAGCAAAGTG TATAGCCAGAGCAAG AACATCCCCCTGTCG  M  T  G  Y  G   S  H  S  K  V   Y  S  Q  S  K   N  I  P  L  S  CAGCCAGCCACCACA ACCGTCAGCACCTCC TTGCCGGTCCCAAAC CCAAGCCTACCTTAC  Q  P  A  T  T   T  V  S  T  S   L  P  V  P  N   P  S  L  P  Y  GAGCAGACCATCGTC TTCCCAGGAAGCACC GGGCACATCGTGGTC ACCTCAGCAAGCAGC  E  Q  T  I  V   F  P  G  S  T   G  H  I  V  V   T  S  A  S  S  ACTTCTGTCACCGGG CAAGTCCTCGGCGGA CCACACAACCTAATG CGTCGAAGCACTGTG  T  S  V  T  G   Q  V  L  G  G   P  H  N  L  M   R  R  S  T  V  AGCCTCCTTGATACC TACCAAAAATGTGGA CTCAAGCGTAAGAGC GAGGAGATCGAGAAC  S  L  L  D  T   Y  Q  K  C  G   L  K  R  K  S   E  E  I  E  N  ACAAGCAGCGTGCAG ATCATCGAGGAGCAT CCACCCATGATTCAG AATAATGCAAGCGGG  T  S  S  V  Q   I  I  E  E  H   P  P  M  I  Q   N  N  A  S  G  GCCACTGTCGCCACT GCCACCACGTCTACT GCCACCTCCAAAAAC AGCGGCTCCAACAGC  A  T  V  A  T   A  T  T  S  T   A  T  S  K  N   S  G  S  N  S  GAGGGCGACTATCAG CTGGTGCAGCATGAG GTGCTGTGCTCCATG ACCAACACCTACGAG  E  G  D  Y  Q   L  V  Q  H  E   V  L  C  S  M   T  N  T  Y  E  GTCTTAGAGTTCTTG GGCCGAGGGACGTTT GGGCAAGTGGTCAAG TGCTGGAAACGGGGC  V  L  E  F  L   G  R  G  T  F   G  Q  V  V  K   C  W  K  R  G  ACCAATGAGATCGTA GCCATCAAGATCCTG AAGAACCACCCATCC TATGCCCGACAAGGT  T  N  E  I  V   A  I  K  I  L   K  N  H  P  S   Y  A  R  Q  G  CAGATTGAAGTGAGC ATCCTGGCCCGGTTG AGCACGGAGAGTGCC GATGACTATAACTTC  Q  I  E  V  S   I  L  A  R  L   S  T  E  S  A   D  D  Y  N  F  GTCCGGGCCTACGAA TGCTTCCAGCACAAG AACCACACGTGCTTG GTCTTCGAGATGTTG  V  R  A  Y  E   C  F  Q  H  K   N  H  T  C  L   V  F  E  M  L  GAGCAGAACCTCTAT GACTTTCTGAAGCAA AACAAGTTTAGCCCC TTGCCCCTCAAATAC  E  Q  N  L  Y   D  F  L  K  Q   N  K  F  S  P   L  P  L  K  Y  ATTCGCCCAGTTCTC CAGCAGGTAGCCACA GCCCTGATGAAACTC AAAAGCCTAGGTCTT  I  R  P  V  L   Q  Q  V  A  T   A  L  M  K  L   K  S  L  G  L  ATCCACGCTGACCTC AAACCAGAAAACATC ATGCTGGTGGATCCA TCTAGACAACCATAC  I  H  A  D  L   K  P  E  N  I   M  L  V  D  P   S  R  Q  P  Y  AGAGTCAAGGTCATC GACTTTGGTTCAGCC AGCCACGTCTCCAAG GCTGTGTGCTCCACC  R  V  K  V  I   D  F  G  S  A   S  H  V  S  K   A  V  C  S  T  TACTTGCAGTCCAGA TATTACAGGGCCCCT GAGATCATCCTTGGT TTACCATTTTGTGAG  Y  L  Q  S  R   Y  Y  R  A  P   E  I  I  L  G   L  P  F  C  E  GCAATTGACATGTGG TCCCTGGGCTGTGTT ATTGCAGAATTGTTC CTGGGTTGGCCGTTA  A  I  D  M  W   S  L  G  C  V   I  A  E  L  F   L  G  W  P  L  TATCCAGGAGCTTCG GAGTATGATCAGATT CGGTATATTTCACAA ACACAGGGTTTGCCT  Y  P  G  A  S   E  Y  D  Q  I   R  Y  I  S  Q   T  Q  G  L  P  GCTGAATATTTATTA AGCGCCGGGACAAAG ACAACTAGGTTTTTC AACCGTGACACGGAC  A  E  Y  L  L   S  A  G  T  K   T  T  R  F  F   N  R  D  T  D  TCACCATATCCTTTG TGGAGACTGAAGACA CCAGATGACCATGAA GCAGAGACAGGGATT  S  P  Y  P  L   W  R  L  K  T   P  D  D  H  E   A  E  T  G  I  AAGTCAAAAGAAGCA AGAAAGTACATTTTC AACTGTTTAGATGAT ATGGCCCAGGTGAAC  K  S  K  E  A   R  K  Y  I  F   N  C  L  D  D   M  A  Q  V  N  ATGACGACAGATTTG GAAGGGAGCGACATG TTGGTAGAAAAGGCT GACCGGCGGGAGTTC  M  T  T  D  L   E  G  S  D  M   L  V  E  K  A   D  R  R  E  F  ATTGACCTGTTGAAG AAGATGCTGACCATT GATGCTGACAAGAGA ATCACTCCAATCGAA  I  D  L  L  K   K  M  L  T  I   D  A  D  K  R   I  T  P  I  E  ACCCTGAACCATCCC TTTGTCACCATGACA CACTTACTCGATTTT CCCCACAGCACACAC  T  L  N  H  P   F  V  T  M  T   H  L  L  D  F   P  H  S  T  H  GTCAAATCATGTTTC CAGAACATGGAGATC TGCAAGCGTCGGGTG AATATGTATGACACG  V  K  S  C  F   Q  N  M  E  I   C  K  R  R  V   N  M  Y  D  T  GTGAACCAGAGCAAA ACCCCTTTCATCACG CACGTGGCCCCCAGC ACGTCCACCAACCTG  V  N  Q  S  K   T  P  F  I  T   H  V  A  P  S   T  S  T  N  L  ACCATGACCTTTAAC AACCAGCTGACCACT GTCCACAACCAGGCT CCCTCCTCTACCAGT  T  M  T  F  N   N  Q  L  T  T   V  H  N  Q  A   P  S  S  T  S  GCCACTATTTCCTTA GCCAATCCCGAAGTC TCCATACTAAACTAC CCATCTACACTCTAC  A  T  I  S  L   A  N  P  E  V   S  I  L  N  Y   P  S  T  L  Y  CAGCCCTCAGCGGCA TCCATGGCTGCAGTG GCCCAGCGGAGCATG CCCCTGCAGACAGGA  Q  P  S  A  A   S  M  A  A  V   A  Q  R  S  M   P  L  Q  T  G  ACAGCCCAGATTTGT GCCCGGCCTGACCCG TTCCAGCAAGCTCTC ATCGTGTGTCCCCCC  T  A  Q  I  C   A  R  P  D  P   F  Q  Q  A  L   I  V  C  P  P  GGCTTCCAAGGCTTG CAGGCCTCTCCCTCT AAGCACGCTGGCTAC TCGGTGCGAATGGAA  G  F  Q  G  L   Q  A  S  P  S   K  H  A  G  Y   S  V  R  M  E  AATGCAGTTCCCATC GTCACTCAAGCCCCA GGAGCTCAGCCTCTT CAGATCCAACCAGGT  N  A  V  P  I   V  T  Q  A  P   G  A  Q  P  L   Q  I  Q  P  G  CTGCTTGCCCAGCAG GCTTGGCCAAGTGGG ACCCAGCAGATCCTG CTTCCCCCAGCATGG  L  L  A  Q  Q   A  W  P  S  G   T  Q  Q  I  L   L  P  P  A  W  CAGCAACTGACTGGA GTGGCCACCCACACA TCAGTGCAGCATGCC ACCGTGATTCCCGAG  Q  Q  L  T  G   V  A  T  H  T   S  V  Q  H  A   T  V  I  P  E  ACCATGGCAGGCACC CAGCAGCTGGCGGAC TGGAGAAATACGCAT GCTCACGGAAGCCAT  T  M  A  G  T   Q  Q  L  A  D   W  R  N  T  H   A  H  G  S  H  TATAATCCCATCATG CAGCAGCCTGCACTA TTGACCGGTCATGTG ACCCTTCCAGCAGCA  Y  N  P  I  M   Q  Q  P  A  L   L  T  G  H  V   T  L  P  A  A  CAGCCCTTAAATGTG GGTGTGGCCCACGTG ATGCGGCAGCAGCCA ACCAGCACCACCTCC  Q  P  L  N  V   G  V  A  H  V   M  R  Q  Q  P   T  S  T  T  S  TCCCGGAAGAGTAAG CAGCACCAGTCATCT GTGAGAAATGTCTCC ACCTGTGAGGTGTCC  S  R  K  S  K   Q  H  Q  S  S   V  R  N  V  S   T  C  E  V  S  TCCTCTCAGGCCATC AGCTCCCCACAGCGA TCCAAGCGTGTCAAG GAGAACACACCTCCC  S  S  Q  A  I   S  S  P  Q  R   S  K  R  V  K   E  N  T  P  P  CGCTGTGCCATGGTG CACAGTAGCCCGGCC TGCAGCACCTCGGTC ACCTGTGGGTGGGGC  R  C  A  M  V   H  S  S  P  A   C  S  T  S  V   T  C  G  W  G  GACGTGGCCTCCAGC ACCACCCGGGAACGG CAGCGGCAGACAATT GTCATTCCCGACACT  D  V  A  S  S   T  T  R  E  R   Q  R  Q  T  I   V  I  P  D  T  CCCAGCCCCACGGTC AGCGTCATCACCATC AGCAGTGACACGGAC GAGGAGGAGGAACAG  P  S  P  T  V   S  V  I  T  I   S  S  D  T  D   E  E  E  E  Q  AAACACGCCCCCACC AGCACTGTCTCCAAG CAAAGAAAAAACGTC ATCAGCTGTGTCACA  K  H  A  P  T   S  T  V  S  K   Q  R  K  N  V   I  S  C  V  T  GTCCACGACTCCCCC TACTCCGACTCCTCC AGCAACACCAGCCCC TACTCCGTGCAGCAG  V  H  D  S  P   Y  S  D  S  S   S  N  T  S  P   Y  S  V  Q  Q  CGTGCTGGGCACAAC AATGCCAATGCCTTT GACACCAAGGGGAGC CTGGAGAATCACTGC  R  A  G  H  N   N  A  N  A  F   D  T  K  G  S   L  E  N  H  C  ACGGGGAACCCCCGA ACCATCATCGTGCCA CCCCTGAAAACCCAG GCCAGCGAAGTATTG  T  G  N  P  R   T  I  I  V  P   P  L  K  T  Q   A  S  E  V  L  GTGGAGTGTGATAGC CTGGTGCCAGGTAAT TTGGGGCCAGGACAG GGCAGGAACCTCTCC  V  E  C  D  S   L  V  P  G  N   L  G  P  G  Q   G  R  N  L  S  CTGGAGAGTGGTTTT CCTGCTTTTCTGCTG CTAGAAATGTTGCTG TATGGGAGCTAG  L  E  S  G  F   P  A  F  L  L   L  E  M  L  L   Y  G  S  * |
| --- | --- |

^EXON 12 in yellow.

^^EXON 13b in green.

**3. HIPK2 Isoform 3 and isoform 1 sequence alignment**

CLUSTAL 2.1 multiple sequence alignment

Isoform1 MAPVYEGMASHVQVFSPHTLQSSAFCSVKKLKIEPSSNWDMTGYGSHSKVYSQSKNIPLS

Isoform3 MAPVYEGMASHVQVFSPHTLQSSAFCSVKKLKIEPSSNWDMTGYGSHSKVYSQSKNIPLS

************************************************************

Isoform1 QPATTTVSTSLPVPNPSLPYEQTIVFPGSTGHIVVTSASSTSVTGQVLGGPHNLMRRSTV

Isoform3 QPATTTVSTSLPVPNPSLPYEQTIVFPGSTGHIVVTSASSTSVTGQVLGGPHNLMRRSTV

************************************************************

Isoform1 SLLDTYQKCGLKRKSEEIENTSSVQIIEEHPPMIQNNASGATVATATTSTATSKNSGSNS

Isoform3 SLLDTYQKCGLKRKSEEIENTSSVQIIEEHPPMIQNNASGATVATATTSTATSKNSGSNS

************************************************************

Isoform1 EGDYQLVQHEVLCSMTNTYEVLEFLGRGTFGQVVKCWKRGTNEIVAIKILKNHPSYARQG

Isoform3 EGDYQLVQHEVLCSMTNTYEVLEFLGRGTFGQVVKCWKRGTNEIVAIKILKNHPSYARQG

************************************************************

Isoform1 QIEVSILARLSTESADDYNFVRAYECFQHKNHTCLVFEMLEQNLYDFLKQNKFSPLPLKY

Isoform3 QIEVSILARLSTESADDYNFVRAYECFQHKNHTCLVFEMLEQNLYDFLKQNKFSPLPLKY

************************************************************

Isoform1 IRPVLQQVATALMKLKSLGLIHADLKPENIMLVDPSRQPYRVKVIDFGSASHVSKAVCST

Isoform3 IRPVLQQVATALMKLKSLGLIHADLKPENIMLVDPSRQPYRVKVIDFGSASHVSKAVCST

************************************************************

Isoform1 YLQSRYYRAPEIILGLPFCEAIDMWSLGCVIAELFLGWPLYPGASEYDQIRYISQTQGLP

Isoform3 YLQSRYYRAPEIILGLPFCEAIDMWSLGCVIAELFLGWPLYPGASEYDQIRYISQTQGLP

************************************************************

Isoform1 AEYLLSAGTKTTRFFNRDTDSPYPLWRLKTPDDHEAETGIKSKEARKYIFNCLDDMAQVN

Isoform3 AEYLLSAGTKTTRFFNRDTDSPYPLWRLKTPDDHEAETGIKSKEARKYIFNCLDDMAQVN

************************************************************

Isoform1 MTTDLEGSDMLVEKADRREFIDLLKKMLTIDADKRITPIETLNHPFVTMTHLLDFPHSTH

Isoform3 MTTDLEGSDMLVEKADRREFIDLLKKMLTIDADKRITPIETLNHPFVTMTHLLDFPHSTH

************************************************************

Isoform1 VKSCFQNMEICKRRVNMYDTVNQSKTPFITHVAPSTSTNLTMTFNNQLTTVHNQAPSSTS

Isoform3 VKSCFQNMEICKRRVNMYDTVNQSKTPFITHVAPSTSTNLTMTFNNQLTTVHNQAPSSTS

************************************************************

Isoform1 ATISLANPEVSILNYPSTLYQPSAASMAAVAQRSMPLQTGTAQICARPDPFQQALIVCPP

Isoform3 ATISLANPEVSILNYPSTLYQPSAASMAAVAQRSMPLQTGTAQICARPDPFQQALIVCPP

************************************************************

Isoform1 GFQGLQASPSKHAGYSVRMENAVPIVTQAPGAQPLQIQPGLLAQQAWPSGTQQILLPPAW

Isoform3 GFQGLQASPSKHAGYSVRMENAVPIVTQAPGAQPLQIQPGLLAQQAWPSGTQQILLPPAW

************************************************************

Isoform1 QQLTGVATHTSVQHATVIPETMAGTQQLADWRNTHAHGSHYNPIMQQPALLTGHVTLPAA

Isoform3 QQLTGVATHTSVQHATVIPETMAGTQQLADWRNTHAHGSHYNPIMQQPALLTGHVTLPAA

************************************************************

Isoform1 QPLNVGVAHVMRQQPTSTTSSRKSKQHQSSVRNVSTCEVSSSQAISSPQRSKRVKENTPP

Isoform3 QPLNVGVAHVMRQQPTSTTSSRKSKQHQSSVRNVSTCEVSSSQAISSPQRSKRVKENTPP

************************************************************

Isoform1 RCAMVHSSPACSTSVTCGWGDVASSTTRERQRQTIVIPDTPSPTVSVITISSDTDEEEEQ

Isoform3 RCAMVHSSPACSTSVTCGWGDVASSTTRERQRQTIVIPDTPSPTVSVITISSDTDEEEEQ

************************************************************

Isoform1 KHAPTSTVSKQRKNVISCVTVHDSPYSDSSSNTSPYSVQQRAGHNNANAFDTKGSLENHC

Isoform3 KHAPTSTVSKQRKNVISCVTVHDSPYSDSSSNTSPYSVQQRAGHNNANAFDTKGSLENHC

************************************************************

Isoform1 TGNPRTIIVPPLKTQASEVLVECDSLVPVNTSHHSSSYKSKSSSNVTSTSGHSSGSSSGA

Isoform3 TGNPRTIIVPPLKTQASEVLVECDSLVPGNLGPGQGRNLSLESGFPAFLLLEMLLYGS--

**************************** * . .. * .*. : . .*

Isoform1 ITYRQQRPGPHFQQQQPLNLSQAQQHITTDRTGSHRRQQAYITPTMAQAPYSFPHNSPSH

Isoform3 ------------------------------------------------------------

Isoform1 GTVHPHLAAAAAAAHLPTQPHLYTYTAPAALGSTGTVAHLVASQGSARHTVQHTAYPASI

Isoform3 ------------------------------------------------------------

Isoform1 VHQVPVSMGPRVLPSPTIHPSQYPAQFAHQTYISASPASTVYTGYPLSPAKVNQYPYI

Isoform3 ----------------------------------------------------------

^EXON 13b coded amino acids in green.

**4. HIPK2 Isoform 3 and alternative spliced sequence alignment**

CLUSTAL 2.1 multiple sequence alignment

Isoform3 MAPVYEGMASHVQVFSPHTLQSSAFCSVKKLKIEPSSNWDMTGYGSHSKVYSQSKNIPLS

Alternative -------MASHVQVFSPHTLQSSAFCSVKKLKIEPSSNWDMTGYGSHSKVYSQSKNIPLS

*****************************************************

Isoform3 QPATTTVSTSLPVPNPSLPYEQTIVFPGSTGHIVVTSASSTSVTGQVLGGPHNLMRRSTV

Alternative QPATTTVSTSLPVPNPSLPYEQTIVFPGSTGHIVVTSASSTSVTGQVLGGPHNLMRRSTV

************************************************************

Isoform3 SLLDTYQKCGLKRKSEEIENTSSVQIIEEHPPMIQNNASGATVATATTSTATSKNSGSNS

Alternative SLLDTYQKCGLKRKSEEIENTSSVQIIEEHPPMIQNNASGATVATATTFTATSKNSGSNS

************************************************ ***********

Isoform3 EGDYQLVQHEVLCSMTNTYEVLEFLGRGTFGQVVKCWKRGTNEIVAIKILKNHPSYARQG

Alternative EGDYQLSQHEVLCSMTNTYEVSEFLGRGTFGQVVKCWKRGTNEIVAIKILKNHPSYARQG

****** ************** **************************************

Isoform3 QIEVSILARLSTESADDYNFVRAYECFQHKNHTCLVFEMLEQNLYDFLKQNKFSPLPLKY

Alternative QIEVSILARLSTESADDYNFVRAYECFQHKNHTCLVFEMLEQNLYDFLKQNKFSPLPLKY

************************************************************

Isoform3 IRPVLQQVATALMKLKSLGLIHADLKPENIMLVDPSRQPYRVKVIDFGSASHVSKAVCST

Alternative IRPVLQQVATALMKLKSLGLIHADLKPENIMLVDPSRQPYRVKVIDFGSASHVSKAVCST

************************************************************

Isoform3 YLQSRYYRAPEIILGLPFCEAIDMWSLGCVIAELFLGWPLYPGASEYDQIRYISQTQGLP

Alternative YLQSRYYRAPEIILGLPFCEAIDMWSLGCVIAELFLGWPLYPGASEYDQIRYISQTQGLP

************************************************************

Isoform3 AEYLLSAGTKTTRFFNRDTDSPYPLWRLKTPDDHEAETGIKSKEARKYIFNCLDDMAQVN

Alternative AEYLLSAGTKTTRFFNRDTDSPYPLWRLKTPDDHEAETGIKSKEARKYIFNCLDDMAQVN

************************************************************

Isoform3 MTTDLEGSDMLVEKADRREFIDLLKKMLTIDADKRITPIETLNHPFVTMTHLLDFPHSTH

Alternative MTTDLEGSDMLVEKADRREFIDLLKKMLTIDADKRITPIETLNHPFVTMTHLLDFPHSTH

************************************************************

Isoform3 VKSCFQNMEICKRRVNMYDTVNQSKTPFITHVAPSTSTNLTMTFNNQLTTVHNQAPSSTS

Alternative VKSCFQNMEICKRRVNMYDTVNQSKTPFITHVAPSTSTNLTMTFNNQLTTVHNQAPSSTS

************************************************************

Isoform3 ATISLANPEVSILNYPSTLYQPSAASMAAVAQRSMPLQTGTAQICARPDPFQQALIVCPP

Alternative ATISLANPEVSILNYPSTLYQPSAASMAAVAQRSMPLQTGTAQICARPDPFQQALIVCPP

************************************************************

Isoform3 GFQGLQASPSKHAGYSVRMENAVPIVTQAPGAQPLQIQPGLLAQQAWPSGTQQILLPPAW

Alternative GFQGLQASSSKHAGYSVRMENAVPIVTQAPGAQPLQIQPGLLAQQAWPSGNQQILLSPTW

********.*****************************************.*****.*:*

Isoform3 QQLTGVATHTSVQHATVIPETMAGTQQLADWRNTHAHGSHYNPIMQQPALLTGHVTLPAA

Alternative QQLDGVATHTSVQHATVIPETMAGTQQLADWRNTHAHGSHYNPIMQQPALLTGHVTLPAA

*** ********************************************************

Isoform3 QPLNVGVAHVMRQQPTSTTSSRKSKQHQSSVRNVSTCEVSSSQAISSPQRSKRVKENTPP

Alternative QPLNVGVAHVMRQQPTSTTSSRKSKQH---------------------------------

***************************

Isoform3 RCAMVHSSPACSTSVTCGWGDVASSTTRERQRQTIVIPDTPSPTVSVITISSDTDEEEEQ

Alternative ------------------------------------------------------------

Isoform3 KHAPTSTVSKQRKNVISCVTVHDSPYSDSSSNTSPYSVQQRAGHNNANAFDTKGSLENHC

Alternative -------VSKQRKNVISCVTVHDSPYSDSSSNTSPYSVQQRAGHNNANAFDTKGSLENHC

*****************************************************

Isoform3 TGNPRTIIVPPLKTQASEVLVECDSLVPGNLGPGQGRNLSLESGFPAFLLLEMLLYGS

Alternative TGNPRTIIVPPLKTQASEVLVECDSLVPGNLGPGQGRNLSLESGFPAFLLLEMLLYGS

**********************************************************

^EXON 12 coded amino acids in yellow.

^^EXON 13b coded amino acids in green.
